# Homogenizing Estimates of Heritability Among SOLAR-Eclipse, OpenMx, APACE, and FPHI Software Packages in Neuroimaging Data

**DOI:** 10.3389/fninf.2019.00016

**Published:** 2019-03-12

**Authors:** Peter Kochunov, Binish Patel, Habib Ganjgahi, Brian Donohue, Meghann Ryan, Elliot L. Hong, Xu Chen, Bhim Adhikari, Neda Jahanshad, Paul M. Thompson, Dennis Van’t Ent, Anouk den Braber, Eco J. C. de Geus, Rachel M. Brouwer, Dorret I. Boomsma, Hilleke E. Hulshoff Pol, Greig I. de Zubicaray, Katie L. McMahon, Nicholas G. Martin, Margaret J. Wright, Thomas E. Nichols

**Affiliations:** ^1^Maryland Psychiatric Research Center, Department of Psychiatry, University of Maryland School of Medicine, Baltimore, MD, United States; ^2^Department of Statistics, University of Oxford, Oxford, United Kingdom; ^3^Department of Cognitive Neuroscience, Maastricht University, Maastricht, Netherlands; ^4^Imaging Genetics Center, Keck School of Medicine of USC, Marina del Rey, CA, United States; ^5^Department of Biological Psychology, VU University, Amsterdam, Netherlands; ^6^Brain Center Rudolf Magnus, Department of Psychiatry, University Medical Center Utrecht, Utrecht, Netherlands; ^7^Faculty of Health, and Institute of Health and Biomedical Innovation, Queensland University of Technology (QUT), Brisbane, QLD, Australia; ^8^Centre for Advanced Imaging, University of Queensland, Brisbane, QLD, Australia; ^9^QIMR Berghofer Medical Research Institute, Brisbane, QLD, Australia; ^10^Queensland Brain Institute, University of Queensland, Brisbane, QLD, Australia; ^11^Big Data Institute, University of Oxford, Oxford, United Kingdom

**Keywords:** DTI, heritability, imaging genetics, reproducability, genetics, population, computational methods

## Abstract

Imaging genetic analyses use heritability calculations to measure the fraction of phenotypic variance attributable to additive genetic factors. We tested the agreement between heritability estimates provided by four methods that are used for heritability estimates in neuroimaging traits. SOLAR-Eclipse and OpenMx use iterative maximum likelihood estimation (MLE) methods. Accelerated Permutation inference for ACE (APACE) and fast permutation heritability inference (FPHI), employ fast, non-iterative approximation-based methods. We performed this evaluation in a simulated twin-sibling pedigree and phenotypes and in diffusion tensor imaging (DTI) data from three twin-sibling cohorts, the human connectome project (HCP), netherlands twin register (NTR) and BrainSCALE projects provided as a part of the enhancing neuro imaging genetics analysis (ENIGMA) consortium. We observed that heritability estimate may differ depending on the underlying method and dataset. The heritability estimates from the two MLE approaches provided excellent agreement in both simulated and imaging data. The heritability estimates for two approximation approaches showed reduced heritability estimates in datasets with deviations from data normality. We propose a data homogenization approach (implemented in solar-eclipse; www.solar-eclipse-genetics.org) to improve the convergence of heritability estimates across different methods. The homogenization steps include consistent regression of any nuisance covariates and enforcing normality on the trait data using inverse Gaussian transformation. Under these conditions, the heritability estimates for simulated and DTI phenotypes produced converging heritability estimates regardless of the method. Thus, using these simple suggestions may help new heritability studies to provide outcomes that are comparable regardless of software package.

## Introduction

Reproducibility is the cornerstone of scientific research. Recent reports on low reproducibility in biomedical research are raising concerns that have to be addressed within the scientific community (Ioannidis, 2014). The emerging field of imaging genetics is not immune to these challenges^1^. Imaging genetics applies modern statistical genetics methods to quantitative phenotypes extracted from high dimensional neuroimaging modalities and has to address replication challenges in both imaging and genetic domains (Thompson et al., 2010). Challenges in replication include low statistical power, complexity of analysis, large number of dependent variables, statistical complexity, and differences in the analysis approaches and software (Meyer-Lindenberg et al., 2008; Collins and Tabak, 2014). All these challenges apply to imaging genetics studies. Imaging genetic studies look for factors that typically explain a small proportion of variance (<1%) and may require a large sample sizes (*N* = 1,000–100,000) to be statistically powerful (Thompson et al., 2014). Imaging genetic studies employ complex analyses involving both imaging and genetic specialized analysis software (Meyer-Lindenberg et al., 2008). We tested the agreement between heritability estimates provided by four methods that are used for heritability estimates in neuroimaging traits. We demonstrated that the heritability estimates may vary by method and sample and propose a way to homogenize the outcomes.

The incomplete description of methods and low statistical power are the two chief factors that are likely contributing to the lack of reproducibility in imaging genetics studies (Collins and Tabak, 2014). Imaging genetic studies combine methods from both imaging and genetic disciplines. These studies require software for extraction of imaging phenotypes and software for genetic analyses of imaging traits, each having individual operating characteristics. For example, the outputs of imaging and genetic software may differ between versions of the same analysis software and even with the same version of software on different operating systems (Gronenschild et al., 2012). Imaging genetic analyses may also suffer from low power because the contribution from common variations in genome to phenotypic variability is typically small (~0.1%), thus requiring large samples to achieve significance and obtain reproducible results (Flint and Munafò, 2013). This further underscores the need for a careful study of the potential biases among different software analysis tools. These methodological biases may lead to challenges to replicate imaging genetic findings if in-kind imaging or genetic software is used during replication.

To address method-related biases, large consortia such as enhancing neuro imaging genetic meta analyses (ENIGMA) have developed standardized multi-site phenotype extraction and genetic analyses pipelines. In this manuscript, we consider the impact of analysis method for the estimation of heritability. We compared four approaches: two commonly used genetic analysis packages (SOLAR-Eclipse and OpenMx), and two recently developed accelerated heritability estimation methods [accelerated permutation inference for ACE (APACE), and fast permutation heritability inference (FPHI)]. These packages use the same variance component model and definition of heritability, but use different numerical methods and data preprocessing steps to calculate the proportion of variance attributed to additive genetic factors. We performed this study to (A) analyze if heritability estimates derived by the four packages’ analyses are comparable to one another and; (B) develop a homogenization approach that minimizes the variability in heritability estimates across the four packages.

We performed these analyses using two datasets: a simulated—with known additive genetic contribution and an experimental—consisting of fractional anisotropy (FA) measurements collected in twins and siblings by three independent studies. FA is the most commonly analyzed scalar parameter extracted from diffusion tensor imaging (DTI; Basser et al., 1994; Basser and Pierpaoli, 1996) and is a sensitive index of fiber coherence, myelination levels, and axonal integrity (Thomason and Thompson, 2011). FA values are under a strong genetic control (Geng et al., 2012; Jahanshad et al., 2013; Shen et al., 2014). Individual differences in FA values are predictive of cognitive performance (Kochunov et al., 2016, 2017) and it is a promising phenotype for multiple neuropsychological disorder including schizophrenia (Friedman et al., 2008; Pérez-Iglesias et al., 2010; Alba-Ferrara and de Erausquin, 2013; Kochunov et al., 2013; Mandl et al., 2013; Nazeri et al., 2013). All experimental data were processed using the harmonization protocol previously developed by ENIGMA and provided on-line at http://enigma.ini.usc.edu/ongoing/dti-working-group/). This included the use of the ENIGMA protocol for following the QA/QC steps for each site, registration to the ENIGMA-DTI target, extraction of white matter skeleton, followed by extraction of tract-average FA values.

## Materials and Methods

### Heritability Estimation Methods

We evaluated the agreement in quantification of the Additive genetic and Environmental, AE, components of the phenotypic variance in simulated and imaging genetic datasets among four heritability calculation methods. SOLAR-Eclipse^2^ and OpenMx^3^ use the iterative maximum likelihood estimation (MLE) approach to fit quantitative genetics variance components models. The iterative MLE approach is used to determine the parameters that maximize the compatibility between the fitted model and the data. It is a versatile computational approach that produces estimates that are optimally precise asymptotically (Almasy and Blangero, 1998; Blangero et al., 2001). SOLAR-Eclipse is an extensive and flexible imaging genetics analysis software package. SOLAR-Eclipse functions include calculation of heritability, genetic correlation, linkage and genome-wide association analysis (Almasy and Blangero, 1998; Blangero et al., 2001). SOLAR-Eclipse polygenic function uses MLE to perform genetic analyses in the pedigrees of arbitrary size and complexity, including twin-siblings and complex multigenerational family designs. SOLAR-Eclipse is frequently used in imaging genetic studies especially in the multi-site analyses that aggregate measurements across multiple datasets using meta and mega-analyses (Jahanshad et al., 2013; Kochunov et al., 2014, 2015). OpenMx is an extensive and flexible structural equation modeling and path analysis library for [R] software (Boker et al., 2011). OpenMX is frequently used by imaging genetic studies to calculate heritability and genetic correlation in twin-siblings pedigrees (Jahanshad et al., 2010; Bootsman et al., 2016). Like SOLAR-Eclipse, OpenMx uses an iterative MLE method for calculation of heritability parameters.

APACE model and FPHI use statistical approximations to estimate heritability values. APACE uses a regression approach based on the squared differences of twin pairs, a variant of a U-statistic (Chen et al., 2013; Chen, 2014), while FPHI starts with the same likelihood as used in SOLAR-Eclipse but uses a single-step, rather than iterative, optimization (Ganjgahi et al., 2015). This overcomes the main limitation of the MLE-based software: long computational times. The iterative MLE heritability calculations in SOLAR-Eclipse and OpenMx can take ~1 s per trait in a pedigree of 1,000 subjects. Therefore, MLE-based heritability analyses require access to large computational clusters to perform imaging genetic analyses that involve 10^4–^10^6^ voxel-wise traits. The non-iterative estimates from APACE and FPHI offer appreciable (~10^3^) gains in computational efficiency. This allows performing voxel-wise heritability analyses on a single workstation. While APACE is only intended for twin or twin-plus-sibling designs, FPHI can use any kinship structure, like SOLAR-Eclipse.

The four software packages were used to compare additive genetic contribution (heritability) in simulated and experimental data using twin family study designs. For experimental data we used DTI acquisitions from three different studies. The human connectome project (HCP; Van Essen et al., 2012), is a large-scale international collaboration aimed at elucidating the genetic and environmental sources of normal variability within the structural and functional connections of the human brain. The other two twin and sibling datasets were drawn from the ENIGMA project, specifically from the ENIGMA-DTI workgroup whose focus is the analysis of DTI data. The first of these is the netherlands twin register (NTR) that collected DTI data in normally developing adolescent twins and siblings. And the other ENIGMA-DTI source is the Brain Structure and Cognition: an Adolescent Longitudinal Twin Study into Genetic Etiology (BrainSCALE). The BrainSCALE dataset collected DTI data in young adult twins and siblings. Subjects for NTR and BrainSCALE datasets were recruited from the same twin register in Netherlands.

We compare heritability estimates for tract-wise average FA values using ENIGMA-DTI, HCP, and simulated data. FA is a widely used quantitative measure of white matter microstructure (Basser et al., 1994; Basser and Pierpaoli, 1996) calculated from the diffusion tensor model of water diffusion (Thomason and Thompson, 2011). Studies suggest FA is an important biomarker in clinical studies, since it is a sensitive index of white matter integrity in Alzheimer’s disease (Clerx et al., 2012; Teipel et al., 2012), general cognitive function (Penke et al., 2010a,b), and several neurological and psychiatric disorders (Sprooten et al., 2011; Barysheva et al., 2012; Carballedo et al., 2012; Kochunov et al., 2013; Mandl et al., 2013). Overall, our goal was to determine if additive genetic contribution (heritability) is comparable between software packages regardless of the variation in the twin-sibling cohort data. Our hypothesis was that estimates of heritability would be consistent amongst the cohorts, irrespective of the variability in cohort data and software package.

### Simulated Data

A simulated *N* = 1,000 person twin-sibling pedigree with 250 monozygotic (MZ) twins, 250 dizygotic (DZ) twins, and 500 founders (not included in the phenotype file) was created using SOLAR-Eclipse simulate function. SOLAR-Eclipse simulation functionality was also used to produce a data set of 10,000 traits with heritability estimates varied uniformly between 0 and 100%. All simulated traits had normal distribution and did not include effects of covariates.

### Experimental Data

#### Human Connectome Project (HCP)

- Subjects: the cohort contained 481 (194/287 M/F; average age 29.1 ± 3.5) healthy participants of the HCP for whom the scans and data were released in June 2014 (humanconnectome.org) after passing the HCP quality control and assurance standards (Marcus et al., 2013). The participants in the HCP study were recruited from the Missouri Family and Twin Registry, a large population-based study (Van Essen et al., 2012). This release included 117 twin pairs (57 MZ and 60 DZ pairs), and 246 of their siblings. The full set of inclusion and exclusion criteria is detailed elsewhere (Van Essen et al., 2012).
- Imaging: diffusion data was collected at Washington University in St. Louis using a customized Siemens Magnetom Connectome 3-Tesla scanner with a 100 mT/m maximum gradient strength and a 32-channel head coil. Details on the scanner, image acquisition and reconstruction are provided in Ugurbil et al. (2013)^4^. Diffusion data were collected using a single-shot, single refocusing spin-echo, echo-planar imaging sequence with 1.25 mm isotropic spatial resolution (TE/TR = 89.5/5520 ms, FOV = 210 × 180 mm). Three gradient tables of 90 diffusion-weighted directions and six *b* = 0 images each, were collected with right-to-left and left-to-right phase encoding polarities for each of the three diffusion weightings (*b* = 1,000, 2,000, and 3,000 s/mm^2^). The total imaging time for collection of diffusion data was approximately 1 h.

#### Netherlands Twin Register (NTR)

- Subjects: the cohort consisted of 246 adults (93/153 M/F; average age 33.9 ± 10.1, range 19–57), recruited from the NTR and consisted of 72 MZ pairs, 48 DZ pairs, and six siblings. Exclusion criteria consisted of having any metal material in the head, having a pacemaker, a history of any major medical conditions or psychiatric illness (den Braber et al., 2008, 2011, 2012).
- Imaging: DTI data were collected on a 3-Tesla Philips Intera MR scanner (32 diffusion-weighted volumes with different non-collinear diffusion directions with *b*-factor = 1,000 s/mm^2^ and one *b*-factor = 0 s/mm^2^ image, flip angle = 90 degrees; 38 axial slices of 3.0 mm; no slice gap; voxel size, 2.0 × 2.0 × 3.0 mm; FOV = 230 mm; TE = 94 ms; TR = 4,863 ms; no cardiac gating; and total scan duration = 185 s).

#### Brain Structure and Cognition: An Adolescent Longitudinal Twin Study into Genetic Etiology (BrainSCALE)

- Subjects: the sample comprised of 199 children (100M/99F; average age 9.2 ± 0.1, range 9.0–9.6). It included 42 MZ and 57 DZ twin pairs that were recruited from families participating in the BrainSCALE cohort (van Soelen et al., 2012) that were recruited from the NTR (van Beijsterveldt et al., 2013). Exclusion criteria consisted of having any metal material in the head, having a pacemaker, a known history of any major medical condition or psychiatric illness. Zygosity was determined based on DNA polymorphisms, using 8–11 highly polymorphic di-, tri- and tetranucleotide genetic markers and confirmed by genome-wide single nucleotide polymorphism data.
- Imaging: DTI data were collected on a 1.5 Philips Achieva MR scanner (32 diffusion-weighted volumes with different non-collinear diffusion directions with *b*-factor = 1,000 s/mm^2^ and eight diffusion-unweighted volumes with *b*-factor = 0 s/mm^2^; parallel imaging SENSE factor = 2.5; flip angle = 90 degrees; 60 slices of 2.5 mm; no slice gap; 96 × 96 acquisition matrix; reconstruction matrix 128 × 128; FOV = 240 mm; TE = 88 ms; TR = 9,822 ms; two repetitions; no cardiac gating; and total scan duration = 296 s). More information may be found in Brouwer et al. (2010, 2012).

### ENIGMA-DTI Processing

We used ENIGMA-DTI protocol to extract whole-brain and tract-wise average FA values for experimental datasets. These protocols are detailed elsewhere (Jahanshad et al., 2013) and are available online at http://enigma.ini.usc.edu/protocols/dti-protocols/. In brief, FA images from all subjects were non-linearly registered to the ENIGMA-DTI target FA image using FSL’s FNIRT (Smith et al., 2006). This target was created as a minimal deformation target based on images from the participating studies as previously described (Kochunov et al., 2002; Jahanshad et al., 2013). The data were then processed using FSL’s tract-based spatial statistics (TBSS) analytic method (Smith et al., 2006) modified to project individual FA values onto the ENIGMA-DTI skeleton mask. After extracting the skeletonized white matter and the projection of individual FA values, ENIGMA tract-wise regions of interest (ROIs), derived from the Johns Hopkins University (JHU) white matter parcellation atlas available as a part of FSL, were transferred to extract the mean FA across the full skeleton and average FA values for major white matter tracts. The protocol, target brain, ENIGMA-DTI skeleton mask, source code and executables are all publicly available^5^. This protocol was shown to provide highly replicable measurements based on test-rest analyses in human subjects (Acheson et al., 2017; McGuire et al., 2017).

### Inverse Normal Transformation

Multivariate quantitative trait models are sensitive to outliers, skewness, kurtosis and other deviations from normal distribution. Therefore, we consider the use of a rank-based inverse normal transformation to ensure the normal distribution in quantitative traits. For each phenotype, rank values are replaced with the expected ranked values of a standard normal distribution with the same number of observations. While it cannot ensure multivariate normality, it does ensure that each univariate distribution is normal and thus reduces the impact of outliers; for more discussion on this transformation see (Beasley et al., 2009). We implemented inverse normalization in SOLAR-Eclipse as the “polyclass_normalize” functions. This function produces inverse normalized residuals for the trait after regression of all covariates. The output from this function was used for the secondary analyses of the imaging data where we first analyze the raw data and then compare our results after the application of the inverse normal transformation to the residual data.

### Heritability Analysis

Heritability analyses were performed in the simulated and FA traits. Heritability (h^2^) is the proportion of the total phenotypic variance (σ_P_^2^) that can be explained by the genetic effects of genes (σ_g_^2^),

(1) $${\text{h}}^{2}={\text{s}}_{\text{g}}^{2}/{\text{s}}_{\text{P}}^{2}$$

### MLE Based Analysis

SOLAR-Eclipse and OpenMX employ MLE based variance decomposition approach that is an extension of the strategy developed by Amos (1994). The multivariate normal covariance matrix Ω for a pedigree of individuals is given by

(2) $$\Omega =2\cdot \Phi \cdot {\text{s}}_{\text{g}}^{2}+\text{I}\cdot {\text{s}}_{\text{e}}^{2}$$

where Φ is the kinship matrix representing the pair-wise kinship coefficients among related individuals, σ_e_^2^ is the variance due to individual-specific environmental effects, and *I* is an identity matrix (under the assumption that all environmental effects are uncorrelated among family members). Narrow sense heritability is defined as the fraction of phenotypic variance σ_P_^2^ attributable to additive genetic factors. In twin designs a third variance parameter is can be identified and may be added to the model, σ_c_^2^, for the common environment shared by twins and siblings growing up in the same family. This three-parameter model is known as the ACE model, while the two-parameter model (Equation 2) is referred to as the AE model.

The variance parameters are estimated by comparing the observed phenotypic covariance matrix with the covariance matrix predicted by kinship (Almasy and Blangero, 1998). Significance of heritability is tested by comparing the likelihood of the model in which σ_g_^2^ is constrained to zero with that of a model in which σ_g_^2^ is estimated. Twice the difference between the log_e_ likelihoods of these models yields a test statistic, which is asymptotically distributed as a 1/2:1/2 mixture of a *X*^2^ variable with 1 degree-of-freedom and a point mass at zero.

#### The Accelerated Permutation for the ACE Model (APACE)

APACE^6^ uses an approximation technique developed originally for animal genetics studies (Grimes and Harvey, 1980) and is based on the result that squared differences of pair’s of subjects’ data reflect their covariance. Thus, the squared differences among the DZ, MZ and unrelated subjects can be entered into a linear regression model to estimate the variance parameters (Grimes and Harvey, 1980). The speed advantage of APACE over MLE approaches allows a permutation analysis to compute familywise error corrected *P*-values for voxel-wise imaging measures.

#### Fast Permutation Heritability Inference (FPHI)

SOLAR-Eclipse’s iterative MLE approach is accelerated by the use of a data transformation based on the eigenvectors of the kinship matrix Φ (Blangero et al., 2013). This transformation converts the dependent data from related subjects into data that is independent but has heterogeneous-variance. SOLAR-Eclipse uses this simplified model to obtain iterative MLE estimates using linear regressions. The FPHI approach uses the same likelihood and data transformation, but then performs just a single step estimation to produce an asymptotically unbiased estimate (Ganjgahi et al., 2015). The FPNI technique is implemented SOLAR-Eclipse as the CPU and graphics processing unit (GPU) functions. The CPU version of FPHI provides a significant (10^3^) computational acceleration relative to the iterative MLE estimation in SOLAR-Eclipse, while the graphics processing unit (GPU) version further improves this performance (~10^6^) vs. iterative MLE approach.

All analyses with imaging data were conducted with age, sex, age^2^, age × sex, and age^2^ × sex included as covariates.

## Results

### Heritability Analyses—Simulated

**Figure 1:**
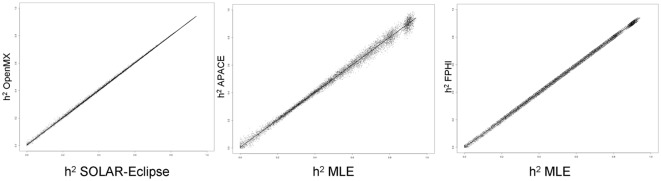
The scatter plot of heritability estimates for 10,000 simulated traits are shown for two ML-based approaches (left). Heritability estimates by two approximation approaches: accelerated permutation inference for ACE (APACE; center) and fast permutation heritability inference (FPHI; right) were plotted vs. the average maximum likelihood estimation (MLE) based values.

Figure 1 shows the scatter plots of four methods using a simulated dataset of heritability values distributed between 0 and 1. The two ML-based methods (SOLAR-Eclipse and OpenMX) showed an excellent agreement (*r* = 0.999, slope = 1.000, intercept = 0.000) with the expected heritability values and with each other (Figure 1). We quantified bias as estimated h^2^ minus true h^2^ and “average spread” as the absolute bias divided by true value (i.e., |estimated − true|/true). In the simulated dataset, the two ML-based methods show zero bias (absolute value bias <10^−6^) and the average spread in heritability estimates of 1.2%. The APACE and FPHI methods showed excellent overall agreement with expected values (APACE: absolute value of bias = 10^−5^, *r* = 0.997, slope = 0.997, intercept = 0.005; FPHI: absolute value of bias = 10^−6^, *r* = 0.998, slope = 0.999, intercept = 0.001). APACE showed significantly higher average spread than the FPHI method: 3.7 vs. 2.2% (*p* = 10^−10^).

### Heritability Analyses—Diffusion Data

**Figure 2:**
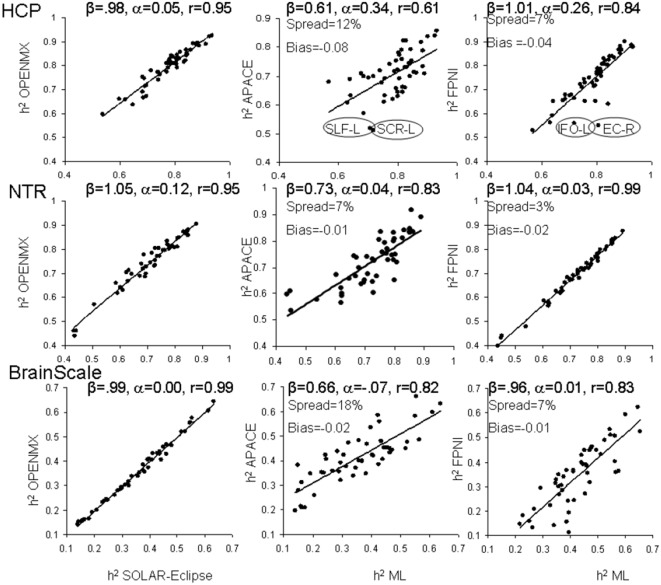
The scatter plot of heritability estimates for 49-regional fractional anisotropy (FA) values calculated by the enhancing neuro imaging genetics analysis (ENIGMA)-diffusion tensor imaging (DTI) pipeline. Heritability estimates for two approximation approaches were plotted vs. the average estimate obtained for two ML-based methods: SOLAR-Eclipse and OpenMX. The lines represent linear regression fit vs. ML-based estimates with slope (β), intercept (α) and Pearson correlation values (r).

The heritability analyses were performed in FA data for 49 tracts in HCP, NTR and BrainScale cohorts using age, sex, age^2^, age × sex, and age^2^ × sex as covariates. The two ML-based method showed excellent agreement in all three datasets (Figure 2). The best agreement was observed in BrainScale data (*r* = 0.99, slope = 0.99, intercept = 0.001). The least agreement (~5% average spread) between two ML-based approaches was observed in HCP (*r* = 0.95, slope = 1.05, intercept = 0.121). Intermediate results were observed in the NTR dataset (*r* = 0.98, slope = 0.98, intercept = 0.055). Hence, we averaged the heritability values produced by the two ML methods to create a “ground truth” reference for the two approximation methods.

The heritability estimates provided by the approximation approaches were more variable among three cohorts (Figure 2). The FPHI showed better accuracy in variance in slopes (*β* = 0.97–1.04) and intercepts (α = 0.01–0.26) vs. APACE (*β* = 0.61–0.73 and intercepts α = −0.07–0.34; Figure 2). Both FPNI and APACE showed a modest negative bias. The highest bias was seen for the HCP cohort (−0.08 and −0.04 for APACE and FPNI, respectively). The bias in NTR and BrainSCALE cohorts was small (−0.01 and −0.02). The spread for FPHI was about half that for APACE (6% vs. 12% for FPHI and APACE, respectively).

### Heritability Analyses—Normalized Diffusion Data

**Figure 3:**
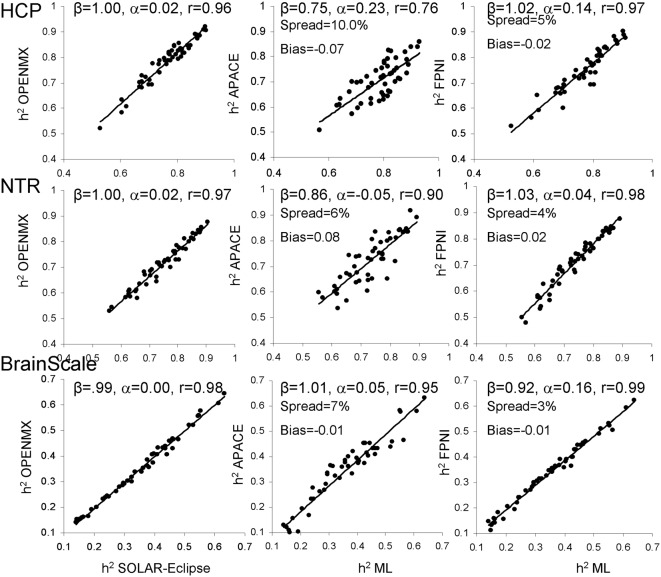
The scatter plot of heritability estimates for 49-regional FA values calculated by ENIGMA-DTI pipeline and then normalized using the trait normalization function in SOLAR-Eclipse. Heritability estimates for two approximation approaches were plotted vs. the average estimate obtained for two ML-based methods: SOLAR-Eclipse and OpenMX. The lines represent linear regression fit vs. ML-based estimates with slope (β), intercept (α) and Pearson correlation values (r).

Next, heritability estimates were calculated on the residual data after inverse normal transformation (Figure 3). Trait normalization improved agreement among the ML-based methods (*r* = 0.96–0.99, slope = 0.99–1.00, intercept = 0.00–0.02; Figure 3).

Trait normalization brought improvements in the agreement between the estimates by two approximation approaches and the average ML-based estimation (Figure 3). APACE method showed improvements in slope (β = 0.75–1.01), intercept (α = −0.05–0.23) and correlation coefficients (*r* = 0.76–0.95), in all three cohorts. For FPHI, the improvements were more subtle and were mainly observed as decrease in bias and spread. The bias for APACE increased for NTR cohort (from −0.01 to 0.08). Both approximation methods showed a 50% improvement in the percentage spread vs. the average ML-based estimate, yet, the % spread for FPHI remained about half that for APACE (4% vs. 7.6% for FPHI and APACE, respectively).

### Analysis of the Disagreement

**Figure 4:**
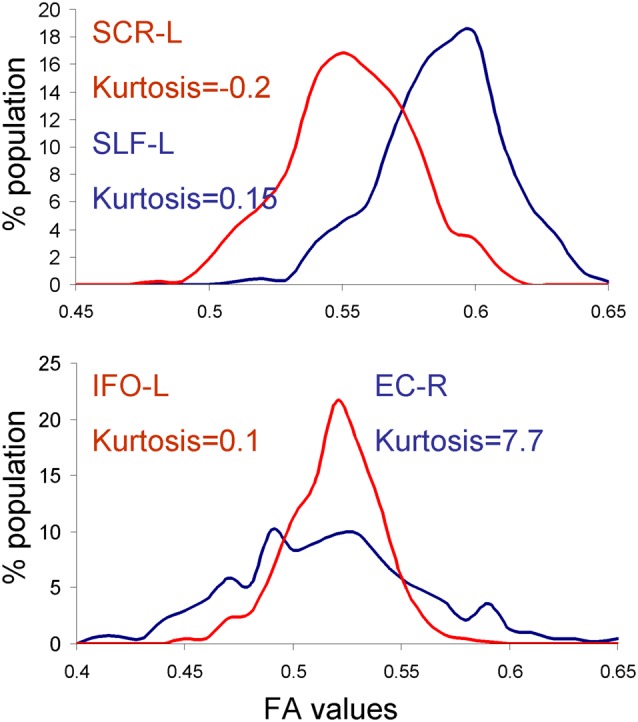
Histograms for the dataset that showed reduced heritability estimates for fast vs. MLE based heritability estimation approaches. APACE showed reduced heritability estimates in superior corona-radiata-right (SCR-L) and SLF-L tracts in the human connectome project (HCP) cohort due to deviations from normal distribution (top panel). FPHI showed reduced heritability estimates in the externalcapsule-right (EC-R) and inferior fronto-occipital tract-left (IFO-L) tracts in the HCP cohort due to the high kurtosis and non-Gaussian shape of the histograms for EC-R and IFO-L, respectively.

We tested the normality of the distribution of the neuroimaging traits using the Shapiro–Wilk method, focusing on the HCP dataset because it had the largest number of subjects. We observed that four traits: the anterior limb of internal capsule-left (ALIC-L), uncinate fasciculus-right (UNC-R), external capsule-right (EC-R) and superior corona radiate-left (CR-L), failed the null hypothesis for normal distribution (*W* > 0.94, *p* < 0.05; Figure 2). However, there was no significant correlation between the deviation from normality or the heritability values for any of the four methods (all *r* < 0.20, all *p* > 0.4). Furthermore, some traits that visibly contributed to dispersion of heritability values, for example the inferior fronto-occipital tract-left (IFO-L) and superior corona-radiata-right (SCR-L; Figure 2), passed the Shapiro–Wilk test (*p* > 0.10). The histograms for SLF-L and SCR-L showed only modest kurtosis (kurtosis = −0.2 and 0.15 for SLF-L and SCR-L), but visibly varied from the normal distribution (Figure 4). The histograms for IFO-L varied visibly from a normal distribution despite having low kurtosis (0.13), while EC-R had high kurtosis (7.7; Figure 4).

## Discussion

We conducted a careful evaluation of four quantitative genetic approaches used by imaging genetic studies to measure heritability—the proportion of variance attributable to the additive genetic factors. Two of the methods (SOLAR-Eclipse and OpenMX) used an iterative MLE approach. Two methods (APCE and FPNI) were developed specifically to accelerate (by 10^3–6^) voxel-wise imaging genetics analyses using fast approximation approaches. We performed the evaluation in a simulated dataset and imaging data from three independent datasets. In the simulated data, we observed an excellent agreement between all heritability estimate approaches. The two MLE approaches accurately replicated the expected heritability values, with the unity slope and near zero intercept and measurement bias. The two approximation techniques likewise showed excellent agreement in the simulated data, with only slight spread (2.2% and 3.7% for FPNI and APACE, respectively). In neuroimaging data, the two MLE approaches produced consistent estimates of heritability for all cohorts. We used the average MLE as the reference measures for approximation techniques because the true additive genetic contribution is unknown (Parisi et al., 2014). In the neuroimaging data, the approximation methods showed deviations from MLE values that varied by the dataset and method. The approximation methods showed the best consistency for NTR and the lowest consistency in the HCP data. *Post hoc* analyses attempted to identify the sources of the dispersion based on the underlying distribution in imaging data. The heritability values were not significantly correlated with Shapiro-Wilk’s *W*-value for any method or dataset (all *r* < 0.2). However, the traits with high dispersion in heritability estimates did show deviations from normality in the underlying dataset. The heritability estimates produced by the FPHI approach were generally closest to that produced by MLE estimates. The agreement among all methods was significantly improved following data normalization approach that ensured normality for quantitative traits. This data normalization approach is now available as a part of SOLAR-Eclipse distribution.

Imaging genetics is a field that combines imaging and genetics—the two disciplines that have greatly advanced neuroscience in recent years. The replication challenges are not unique to this new field and require concerted efforts to address them. The main replication challenges that imaging genetics faces are the complexity of the methods and the low statistical power (Meyer-Lindenberg et al., 2008; Collins and Tabak, 2014). Genetic factors may explain a small proportion of variance that require a sample sizes that are challenging to collect in a single study (*N* = 1,000–100,000; Stein et al., 2010, 2012; Thompson et al., 2014). Yet, imaging genetics approaches have many advantages that should help in overcoming this challenge. Modern MRI offers phenotypic measurements that provide more detailed and quantitative descriptions than disorder diagnostic status or clinical symptoms. Modern MRI phenotypes offer high precision and reproducibility with the inter-session, scan-rescan variability of many common imaging measurements in the range of 1%–5% (Agartz et al., 2001; Kim et al., 2005; Lerch and Evans, 2005; Kochunov and Duff Davis, 2009; Acheson et al., 2017). Therefore, the solution to statistical power is meta-analyses that combine data across multiple studies.

ENIGMA, Cohorts for Heart and Aging Research in Genomic Epidemiology (CHARGE) and other multi-study initiatives aim to overcome the challenge of limited power by performing meta-analytical analyses. In these initiatives, phenotypic and genetic analyses are performed by individual sites and meta-analytical aggregation is used to derive the overall estimates of genetic effects. The main challenge in this approach is overcoming the diversity and complexity of analytical and statistical approaches that may lead to variance in phenotype extractions and estimation of effect sizes (Meyer-Lindenberg et al., 2008; Collins and Tabak, 2014). This complexity exists on both imaging and genetic sides where the difference in analysis software and even versions of software may lead to varying results (Gronenschild et al., 2012). On the phenotype extraction side, ENIGMA provides the standardized pipeline for extraction of homogenized neuroimaging phenotypes across the sites (Jahanshad et al., 2013). Here, we demonstrate the need of homogenized treatments of the traits to avoid erroneous variances at the meta-analytical state.

In our evaluations, we observed excellent agreement between estimates produced by the two MLE-based approaches that were the corner stone of imaging genetic research in the past. The main disadvantage of MLE approaches is the long calculation times associated with the iterative maximization of the likelihood. In imaging genetic studies, up to a million voxel-based imaging traits may be analyzed (Stein et al., 2011), making MLE approaches less practical. Voxel-wise analyses require a permutation-based correction for multiple comparisons because standard multiple comparison approaches are deemed to be too conservative for voxel-wise traits (Nichols and Hayasaka, 2003). Therefore, there is a need for fast and accurate methods to estimate genetic variance where the calculations can be repeated with 10^5–6^ permutations to derive cluster-based significance on the voxel-wise levels. We measured the performance of two such methods (APACE and FPNI) that use approximation to obtain fast inference of genetic variance.

APACE and FPNI use data transformation and approximation fits to accelerate the calculation of genetic parameters. APACE uses a squared difference in phenotype values between pairs of related and unrelated subjects to derive the fraction of variance contributable to the additive genetic variance. This approach is appropriate for twins and siblings pedigree. FPNI uses the eigenvalue decomposition followed by a single step approximation to calculate genetic variance in pedigrees of any complexities. The approximation approaches demonstrated an excellent performance in the simulated dataset where the trait data was normally distributed. However, their performance in the imaging data was less uniform, likely due to sensitivity to noise and violations of the normality assumption.

The two MLE approaches appeared to provide more stable estimates of heritability in datasets with noise and the non-normally distributed traits, while these deviations had a greater impact on the heritability estimates produced by the approximation approaches. In the cases where the trait’s distribution deviated from normality, the heritability values calculated by the approximation techniques deviated from those calculated by ML-based approaches. However, the correlation between heritability values and the deviation of normality (Shapiro-Wilk’s W) was not significant. We explored four cases of visible outliers. Some traits (ALIC-L, UNC-R, EC-R and CR-L) failed assumptions for normality, but other outliers passed normality according to Shapiro-Wilk’s test. We concluded that approximation approaches may be more sensitive to the noise and deviation from data normality and may produce biased heritability estimates even in traits whose distributions pass the standard tests for normality.

We found that the use of inverse normal transformation improved the agreement between ML and approximation-based approaches and resolved the outlier heritability estimates observed in uncorrected data. The inverse normal transformation did not alter the pattern of ML-based estimates: high correlation (*r* > 0.95) was observed for averaged ML-estimates before and after inverse normal transformation. Enforcing normality upon data reduced the dispersion in h^2^ values and improved the average spread for the approximation approaches. This was especially noticeable for FPNI approach where the correlations with ML-estimates became high (*r* > 0.97) for all cohorts.

## Limitation

The ML estimations were used as the reference to compare the performance of approximation-based approaches in the simulated and imaging data. The two ML approaches produced convergent heritability estimates in both simulated and imaging datasets. However, this does not constitute the “ground truth” especially in imaging datasets where ML approaches may be biased despite convergence.

## Conclusion

We have conducted a careful comparison of four heritability estimation methods for imaging data. Based on “ground-truth” simulations, four packages can produce low-bias, low-variance heritability estimates, with ML-based methods understandably performing slightly better than the approximation methods. In real data, the approximation methods exhibit more variability relative to the ML-based methods, but this variability was reduced with the use of a rank-based inverse normal transformation, suggesting that this may be an important tool to maximize inter-method reliability.

## Ethics Statement

This study performed secondary data analyses in anonymized human subjects.

## Author Contributions

PK, BP, HG, BD, MR, XC, NJ, PT and TN designed experiment, performed analyses, and wrote the manuscript. All authors listed have made a substantial, direct and intellectual contribution to the work, and approved it for publication.

## Conflict of Interest Statement

The authors declare that the research was conducted in the absence of any commercial or financial relationships that could be construed as a potential conflict of interest.

## Footnotes

^1^http://www.biorxiv.org/content/early/2017/02/20/107987

^2^www.solar-eclipse-genetics.org

^3^openmx.ssri.psu.edu

^4^http://www.humanconnectome.org/documentation/S500/HCP_S500_Release_Reference_Manual.pdf

^5^https://www.nitrc.org/projects/enigma_dti

^6^http://warwick.ac.uk/tenichols/software/APACE
